# Rat N-ERC/Mesothelin as a Marker for *In Vivo* Screening of Drugs against Pancreas Cancer

**DOI:** 10.1371/journal.pone.0111481

**Published:** 2014-10-27

**Authors:** Katsumi Fukamachi, Masaaki Iigo, Yoshiaki Hagiwara, Koji Shibata, Mitsuru Futakuchi, David B. Alexander, Okio Hino, Masumi Suzui, Hiroyuki Tsuda

**Affiliations:** 1 Department of Molecular Toxicology, Nagoya City University Graduate School of Medical Sciences, Nagoya, Japan; 2 Nanotoxicology Project, Nagoya City University, Nagoya, Japan; 3 Immuno-Biological Laboratories, Fujioka-shi, Gunma, Japan; 4 Division of Surgical Oncology, Department of Surgery, Nagoya University Graduate School of Medicine, Nagoya, Japan; 5 Department of Pathology and Oncology, Juntendo University School of Medicine, Tokyo, Japan; Hormel Institute, University of Minnesota, United States of America

## Abstract

Pancreatic ductal adenocarcinoma (PDA) is a highly lethal disease, which is usually diagnosed in an advanced stage. We have established transgenic rats carrying a mutated K-*ras* gene controlled by Cre/loxP activation. The animals develop PDA which is histopathologically similar to that in humans. Previously, we reported that serum levels of N-ERC/mesothelin were significantly higher in rats bearing PDA than in controls. In the present study, to determine whether serum levels of N-ERC/mesothelin correlated with tumor size, we measured N-ERC/mesothelin levels in rats bearing PDA. Increased serum levels of N-ERC/mesothelin correlated with increased tumor size. This result indicates an interrelationship between the serum level of N-ERC/mesothelin and tumor size. We next investigated the effect of chemotherapy on serum N-ERC/mesothelin levels. Rat pancreatic cancer cells were implanted subcutaneously into the flank of NOD-SCID mice. In the mice treated with 200 mg/kg gemcitabine, tumor weight and the serum level of N-ERC/mesothelin were significantly decreased compared to controls. These results suggest that serum N-ERC/mesothelin measurements might be useful for monitoring response to therapy.

## Introduction

N-ERC/mesothelin is a 31-kDa protein that forms the N-terminal fragment of the full-length 71-kDa ERC/mesothelin protein and is secreted into the blood of mesothelioma patients [1]–[3]. The rat *Erc* (expressed in renal cell carcinoma) gene was identified as a highly expressed gene in renal cell carcinoma of the Eker rat [4], [5]. The human homolog of rat *Erc* is the *Mesothelin*/*megakaryocyte potentiating factor* (*MPF*) gene [6]–[8]. ERC/mesothelin is also expressed in ovary and pancreas carcinoma tissue in humans [9]–[12]. N-ERC/mesothelin is considered to be relevant in monitoring chemotherapeutic response in patients with mesothelioma [13], [14].

Pancreatic ductal adenocarcinoma (PDA) carries the most dismal prognosis of all solid tumors [15]. We have established transgenic rat lines carrying a human H*ras* ^G12V^ (Hras250) [16] or a human K*ras* ^G12V^ (Kras301, Kras327) [17] oncogene in which the expression of the transgene is regulated by the Cre/loxP system. Targeted activation of the transgene is accomplished by injection of a Cre recombinase-carrying adenovirus (AxCANCre) into the pancreatic ducts through the common bile duct. Neoplastic lesions in the transgenic rats exhibit morphological [16], [18] and biological [17], [19]–[21] similarities to those observed in human pancreas lesions. We previously reported that N-ERC/mesothelin is readily detected in the serum of rats bearing pancreas ductal adenocarcinomas [17]. In the present study we report the use of N-ERC/mesothelin as a serum marker for evaluation of the effectiveness of chemotherapy.

## Materials and Methods

### Animals

Male K*ras* ^G12V^ transgenic (Kras301) rats (Jcl:SD rat background) were established as previously reported [17], [18]. Homozygous rats were generated by breeding heterozygous male rats with heterozygous female rats. Homozygous transgenic rats were identified by semi-quantitative PCR and then confirmed by genetic testing. Kras301 rats were bred to obtain viable and fertile homozygous transgenic progeny. Fisher344 (F344/DuCrlCrlj) rats were purchased from Charles River Japan, Inc. (Yokohama, Japan). Sprague-Dawley (SD) rats (Jcl:SD) were obtained from CLEA Japan (Tokyo, Japan). SD (Slc:SD) and Wistar (Slc:Wistar/ST) rats were obtained from Japan SLC, Inc. (Hamamatsu, Japan). The rats were maintained in plastic cages in an air-conditioned room with a 12-h light/12-h dark cycle. All experiments were conducted according to the Guidelines for Animal Experiments of the Nagoya City University Graduate School of Medical Sciences, and approved by the Animal Care and Use Committee of the Nagoya City University Graduate School of Medical Sciences (H21-02, H22M-24).

### Tumor induction and pathological examination

Cre-expressing adenovirus vectors were amplified in HEK293 cells and then purified using Vivapure Adenopack (Vivascience, Hannover, Germany) as previously reported [17]. Pancreas tumors were induced as described previously [16]–[22]. The pancreas was fixed in 4% paraformaldehyde and processed for histological observation. Anti-rat C-ERC/mesothelin (306) was purchased from Immuno-Biological Laboratories (Gunma, Japan), and staining was performed as described previously [17].

### Transplantation of a rat pancreas cell line and treatment with gemcitabine

A pancreas carcinoma cell line (634NOD) was established from a pancreas tumor from an Hras250 rat [17], [23]. 634NOD tumor cells (2×10^6^) were transplanted subcutaneously into 6 week old male NOD-SCID mice (CLEA Japan, Tokyo, Japan). One week later, NOD-SCID mice received gemcitabine (2′, 2′-difluoro deoxycytidine; dFdC) hydrochroride (Eli Lilly Japan K.K., Kobe, Japan) in saline four times within nine days by intraperitoneal injection. Tumors were measured by length (a) and width (b) in millimeters using calipers, and tumor volumes (V) were calculated according to the relationship V = ab^2^/2, where “a” was the longer of the two measurements [24]. Seven weeks after transplantation, the mice were killed under deep isoflurane anesthesia and the tumors were removed and weighed and serum was collected for analysis of N-ERC/mesothelin.

### Serum test

The serum level of rat N-ERC/mesothelin was quantified by ELISA (Code No.27765, Rat N-ERC/mesothelin Assay Kit, IBL, Gunma, Japan) as described previously [25].

## Results

### Serum level of N-ERC/mesothelin in Kras301 rats

**Figure 1:**
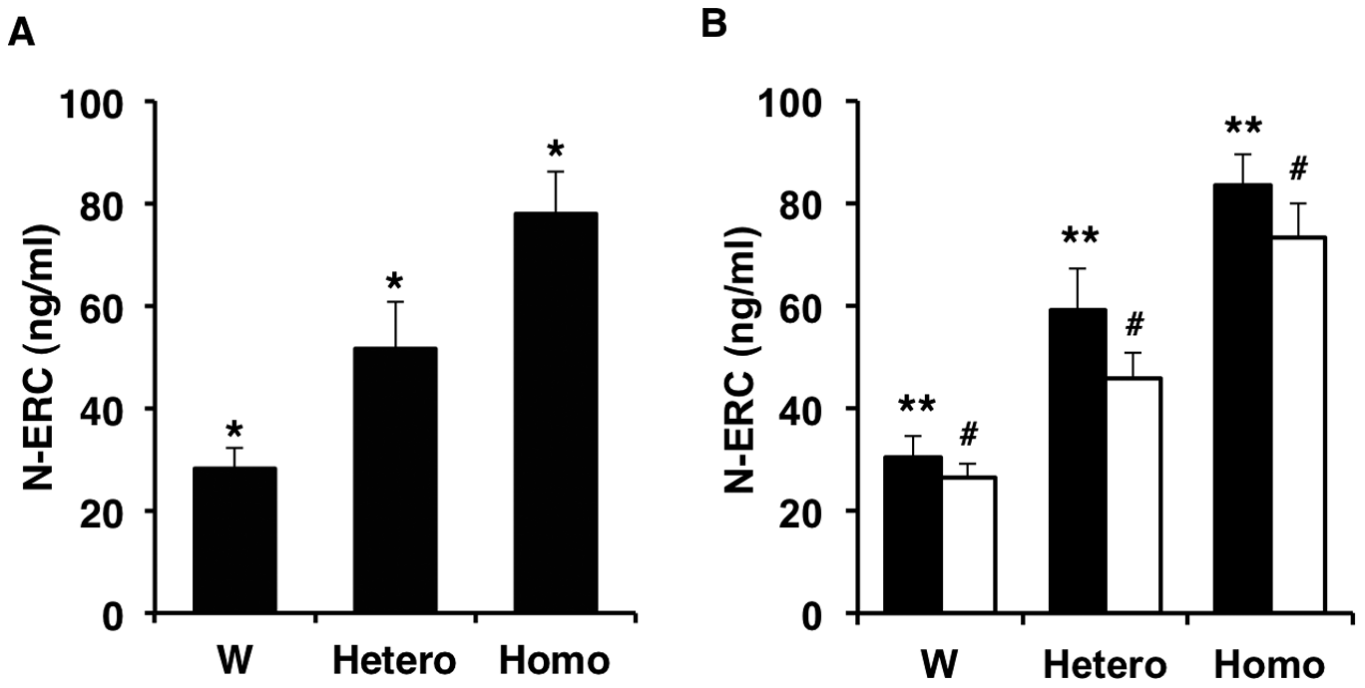
Comparison of serum levels of N-ERC/mesothelin in wild type Jcl:SD (W) and heterozygous (Hetero) and homozygous (Homo) Kras301 rats. Heterozygous Kras301 rats were generated by breeding homozygous Kras301 rats with wild type Jcl:SD rats. (A) Serum levels of N-ERC/mesothelin in wild type Jcl:SD rats and heterozygous and homozygous Kras301 rats. (B) Serum levels of N-ERC/mesothelin in male (closed bar) and female (open bar) wild type Jcl:SD rats and heterozygous and homozygous Kras301 rats. *, ** and #, P<0.0001 compared with each other.

To determine whether the serum level of N-ERC/mesothelin is different in wild type Jcl:SD rats and heterozygous and homozygous Kras301 rats, we assayed N-ERC/mesothelin levels in wild type Jcl:SD rats and heterozygous and homozygous Kras301 rats at the age of 10 weeks: heterozygous F1 rats were generated by breeding homozygous male Kras301 rats with female Jcl:SD rats. There was no significant difference in body weight among the males or the females of the different genotypes (data not shown). The serum levels of N-ERC/mesothelin was significantly different between the three genotypes (P<0.0001): 28.3±4.0 ng/ml (n = 19) in the wild type Jcl:SD rats, 51.5±9.2 ng/ml (n = 19) in the heterozygous Kras301 rats, and 77.9±8.2 ng/ml (n = 13) in the homozygous Kras301 rats (Figure 1A). Furthermore, the serum level of N-ERC/mesothelin in heterozygous F1 rats was clearly higher than the original heterozygous rats [17].

There was no significant difference in serum N-ERC/mesothelin levels between male (30.3±4.2 ng/ml, n = 9) and female (26.4±2.8 ng/ml, n = 10) wild type Jcl:SD rats. In contrast, there was a significant difference in the serum levels of N-ERC/mesothelin between male and female heterozygous Kras301 F1 rats (male, 59.2±8.0 ng/ml, n = 8; female, 45.9±5.0 ng/ml, n = 11: P<0.0001) and homozygous Kras301 rats (male, 83.4±6.2 ng/ml, n = 6; female, 73.2±6.7 ng/ml, n = 7: P<0.05) (Figure 1B).

### Serum level of N-ERC/mesothelin in different rat strains

**Figure 2:**
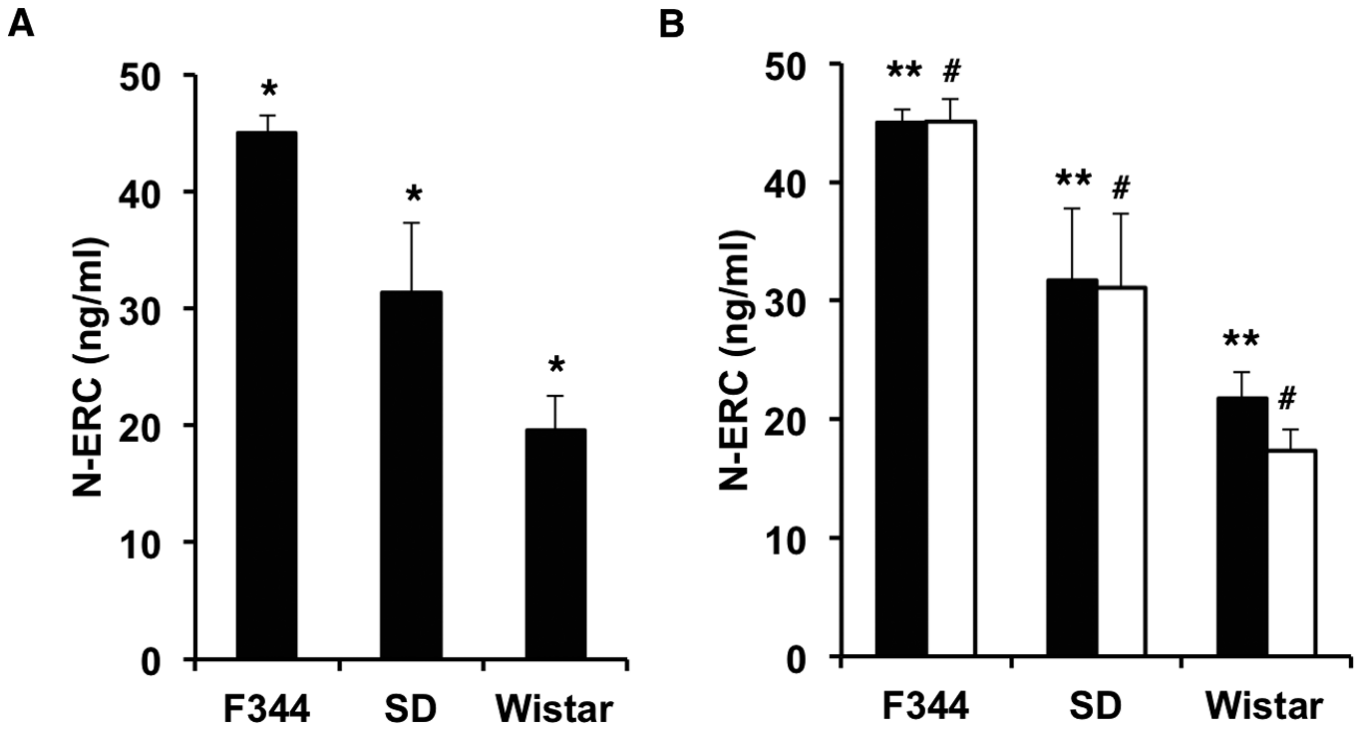
Comparison of serum levels of N-ERC/mesothelin in F344/DuCrlCrlj (F344), Slc:SD (SD) and Slc:Wistar/ST (Wistar) rat strains. (A) Strain differences in the serum levels of N-ERC/mesothelin. (B) Serum levels of N-ERC/mesothelin in males (closed bar) and females (open bar) of the different rat strains. *, P<0.0001; **, P<0.005; #, P<0.0001 compared with each other.

To determine whether the genetic background affected the serum levels of N-ERC/mesothelin, the levels of N-ERC/mesothelin in the serum were measured in three different rat strains: F344/DuCrlCrlj, Slc:Wistar/ST, and Slc:SD. The levels of N-ERC/mesothelin were significantly different between rat strains (P<0.0001) (Figure 2A). The levels of N-ERC/mesothelin were 45.0±1.5 (n = 12) in F344/DuCrlCrlj, 31.4±5.9 (n = 12) in Slc:SD and 19.6±3.0 (n = 12) in Slc:Wistar/ST rats. The levels of N-ERC/mesothelin were also significantly different between the males of the different strains and the females of the different strains (Figure 2B). In the individual strains, there was no significant difference between male (n = 6) and female (n = 6) rats (Figure 2B). Furthermore there was no significant difference between Jcl:SD (n = 19) and Slc:SD (n = 12) rats.

### Serum level of N-ERC/mesothelin correlated with tumor size in rats

**Figure 3:**
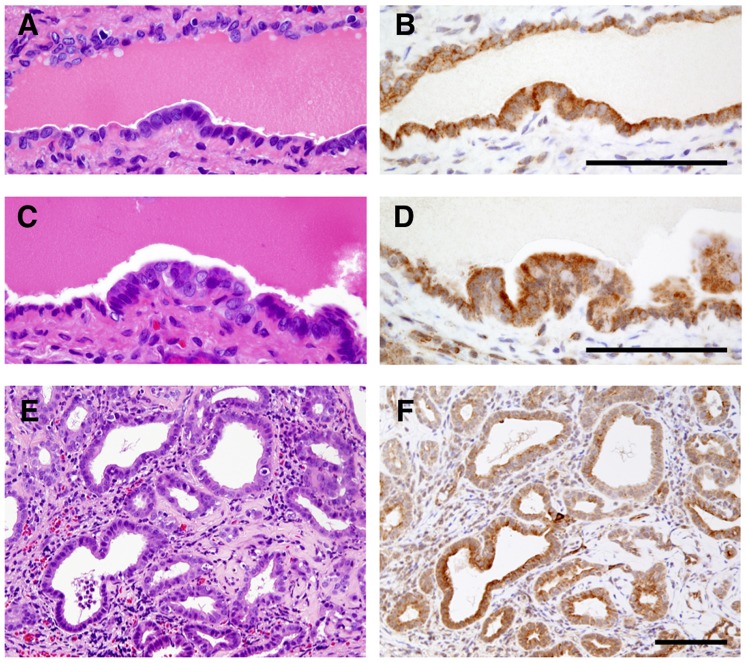
Expression of ERC/mesothelin in pancreatic lesions. Immunostaining of ERC/mesothelin in PanIN1 (A and B), PanIN2 (C and D) and PDA (E and F) lesions. A, C, E, H&E staining; B, D, F, ERC/mesothelin staining. Bars = 100 µm.

To determine whether serum levels of N-ERC/mesothelin correlated with tumor size, we assayed N-ERC/mesothelin levels in homozygous Kras301 rats bearing pancreas ductal adenocarcinomas. Adult male homozygous Kras301 rats were killed 4 weeks after injection of recombinant AxCANCre into the pancreatic duct via the common bile duct: see Fukamachi et al. 2013 [22] for a detailed description of AxCANCre-mediated activation of the Kras oncogene in Kras301 rats. Many grossly visible whitish tumor nodules were observed throughout the pancreas in the transgenic rats. Histological examination showed that these nodules were adenocarcinomas, as has been reported previously [16], [17]. Neoplastic lesions were not found in any other organs. Immunohistochemical studies showed higher expression of ERC/mesothelin in carcinoma lesions compared to surrounding normal tissue, as has been reported previously [17]. Furthermore, immunohistochemical studies showed ERC/mesothelin was also positively expressed in pancreatic intraepithelial neoplasia (PanIN) lesions (Figure 3).

**Figure 4:**
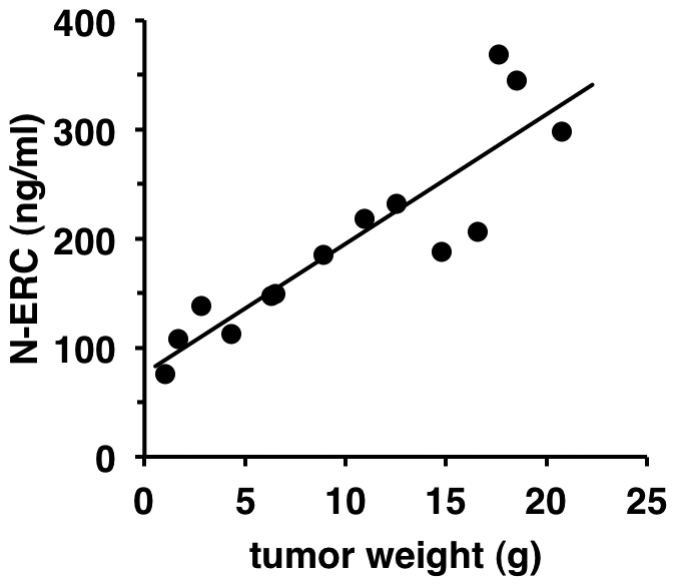
Correlation between tumor weight and serum level of N-ERC/mesothelin. The serum level of N-ERC/mesothelin in homozygous Kras301 rats bearing pancreas ductal carcinomas. The serum level of N-ERC/mesothelin correlated with increased tumor weight (R = 0.897).

An increase in the serum level of N-ERC/mesothelin correlated with increased tumor size (R = 0.89665, P<0.0001, n = 14) (Figure 4). This result indicates an interrelationship between the serum level of N-ERC/mesothelin and tumor size in these rats.

### N-ERC/mesothelin as a marker of the chemotherapeutic effect of an anti-cancer drug

**Figure 5:**
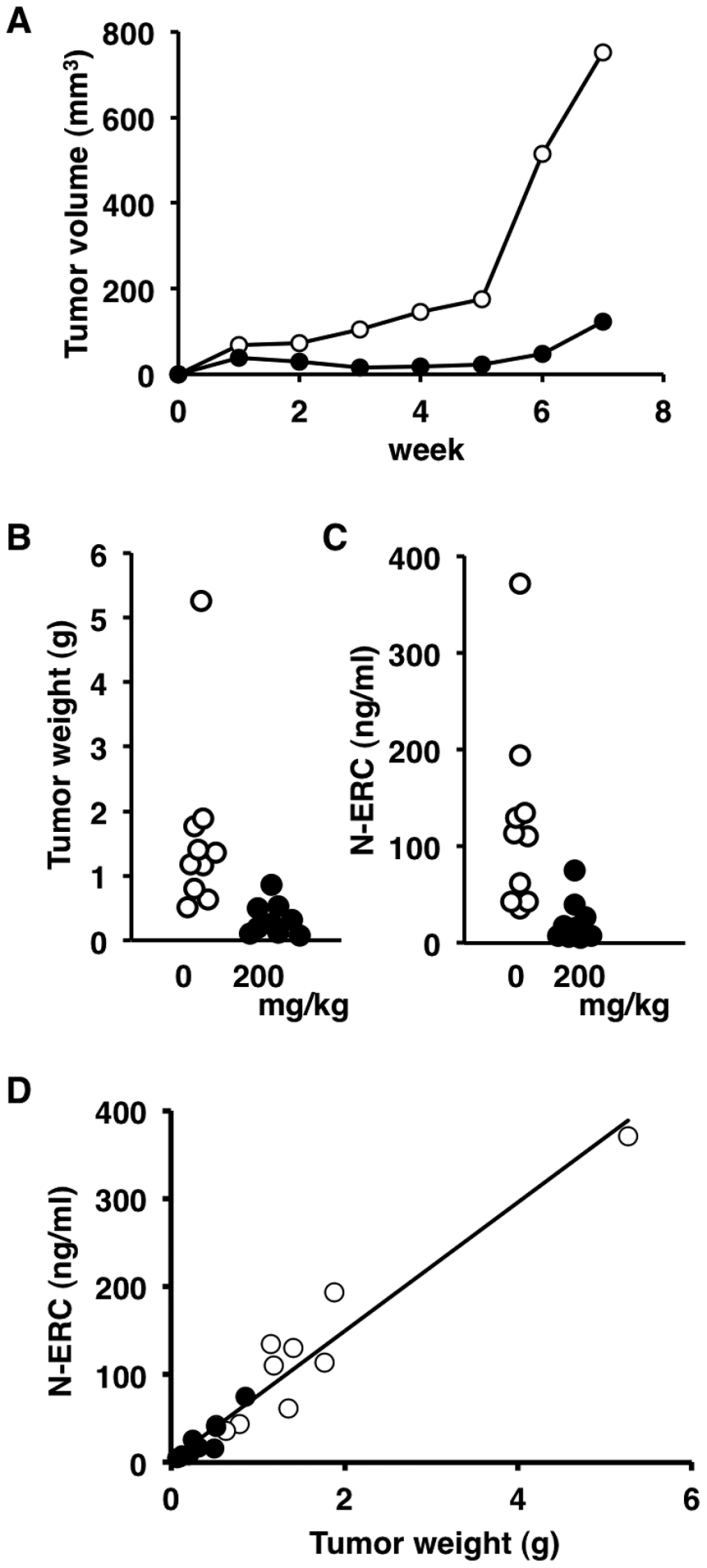
Effect of gemcitabine in NOD-SCID mice with transplanted 634NOD cells. Rat pancreas cancer cells (634NOD cells) were implanted subcutaneously into the flank of NOD-SCID mice. After 1 week the mice were administered gemcitabine (0 or 200 mg/kg, i.p.) four times within nine days by intraperitoneal injection. (A) Periodic observation of tumors in NOD-SCID mice treated with gemcitabine (0 mg/kg, open circles; 200 mg/kg, closed circles). (B) The tumor weight and (C) serum level of N-ERC/mesothelin was significantly decreased by treatment with gemcitabine (200 mg/kg, closed circles) compared to control (open circles) (P<0.05, P<0.01 respectively). (D) Correlation of tumor weight and serum levels of N-ERC/mesothelin. The serum level of N-ERC/mesothelin correlated with tumor weight and gemcitabine treatment (R = 0.966). Open circles, 0 mg/kg; closed circles, 200 mg/kg.

We next investigated whether serum N-ERC/mesothelin levels were affected by treatment of pancreas adenocarcinoma with the chemotherapeutic drug gemcitabine. 634NOD cells [17], which were derived from a pancreas ductal adenocarcinoma from an Hras250 rat, were implanted subcutaneously into the flank of NOD-SCID mice. After 1 week the mice were administered gemcitabine (200 mg/kg, i.p.) four times within nine days by intraperitoneal injection. From week 1 to week 7 the tumor size continued to increase in both the control and the gemcitabine treated mice (Figure 5A). Although, due to the wide dispersion of tumor sizes, there was not a significant difference in tumor size between the two groups, the average tumor size was markedly smaller in the gemcitabine-treated mice compared to the control mice from week 3 to week 7 (Figure 5A). At the end of week 7, the mice were sacrificed and the tumors removed and weighed; the tumor weight in the gemcitabine-treated mice (0.33±0.26 g, n = 9) was significantly decreased compared to the control mice (1.60±1.37 g, n = 10) (P<0.05) (Figure 5B). There was no morphological difference between tumors from the gemcitabine-treated and control mice (data not shown). At the end of week 7, the serum level of N-ERC/mesothelin was significantly decreased (P<0.01) in mice treated with gemcitabine (22.4±22.6 ng/ml, n = 9) compared to the control mice (123.7±100.6 ng/ml, n = 10) (Figure 5C). In contrast, neither tumor weight nor serum levels of N-ERC/mesothelin were significantly different from the control (n = 10) in mice treated with 75 (n = 10) or 150 (n = 9) mg/kg gemcitabine (Figure S1). Although due to the wide dispersion of tumor sizes and N-ERC/mesothelin levels, there was not a significant difference in tumor size or N-ERC/mesothelin levels between the mice treated with 75 or 150 mg/kg gemcitabine and the control mice, the average of both values was smaller in the gemcitabine-treated mice compared to the control mice. Thus, the level of serum N-ERC/mesothelin correlated well with the effect of gemcitabine (R = 0.96597, P<0.0001) (Figure 5D).

## Discussion

We have previously reported that the serum level of N-ERC/mesothelin correlated with tumor size in mice with transplanted rat pancreas cancer cells (634NOD cells) [17]. In the present study, in agreement with our previous report, the serum level of N-ERC/mesothelin correlated with pancreas tumor size in rats (Figure 4). These results showed that relative pancreas tumor size could be estimated from the serum level of N-ERC/mesothelin without sacrificing the animals. We also showed that N-ERC/mesothelin in the serum was a biomarker that could be used for monitoring the response to chemotherapy. When mice with tumors derived from transplanted 634NOD cells were treated with gemcitabine, tumor weight and the level of N-ERC/mesothelin in the serum were decreased compared with control. The serum level of N-ERC/mesothelin correlated with the therapeutic effect of the anti-cancer drug (Figure 5). Therefore, N-ERC/mesothelin can be used for quantitative analysis of the therapeutic effect of anti-cancer agents on pancreas cancer in the animal models used in this study.

The serum level of N-ERC/mesothelin in homozygous Kras301 rats was significantly higher than those of wild type rats. The serum level of N-ERC/mesothelin in heterozygous F1 of homozygous rats was also significantly higher than in wild type rats. However, the serum level of N-ERC/mesothelin in the original heterozygous rats were not different from wild type Jcl:SD rats [17]. The homozygous Kras301 rats were maintained by breeding homozygous male and homozygous female rats. It is likely that rats with high levels of N-ERC/mesothelin were accidentally selected in the course of establishing the homozygous Kras301 rats.

The serum level of N-ERC/mesothelin was significantly different among rat strains (F344/DuCrlCrlj, Slc:SD and Slc:Wistar/ST). These results suggest that the genetic background of different rat strains affects the basal level of serum N-ERC/mesothelin. Therefore, for the evaluation of chemotherapeutic responses using serum N-ERC/mesothelin, it is important to use appropriate controls.

N-ERC/mesothelin was detected in the supernatants of cultured human pancreas cancer cells and correlated with the expression levels of ERC/mesothelin [26]. ERC/mesothelin is frequently expressed in ductal carcinoma, but not in normal pancreas [11], [26]–[28]. Thus, ERC/mesothelin expression can be found in both human and rat pancreas ductal adenocarcinoma. However, contrary to expectations, there was no significant difference in serum N-ERC/mesothelin concentrations between pancreas cancer patients and healthy control groups [26]. Therefore, while N-ERC/mesothelin can be used as a serum biomarker for pancreas ductal carcinoma in the animal models used in the present study, it is not suitable for humans.

Currently, there is no effective drug against pancreas cancer; therefore, the use of animal models to test the responsiveness of pancreas cancer to therapy remains of the utmost importance. The use of rat N-ERC/mesothelin as a serum marker lies in its ability to distinguish between rats with or without pancreas cancer without opening the abdominal cavity. Gemcitabine is generally considered to constitute first-line therapy for pancreatic cancer. The maximum tolerated dose of gemcitabine in rats was 50 mg/kg once a week. This dose had no effect on tumor size in Kras301 rats. Therefore, the serum level of N-ERC/mesothelin was not significantly affected in these animals (our unpublished results). Notably, in mice treated with gemcitabine, levels of gemcitabine that did not significantly affect tumor size did not affect the serum level of N-ERC/mesothelin (Figure S1). While gemcitabine alone does not show anti-cancer effects on endogenously developed pancreas cancer in rats (our unpublished result) or in mouse models [29], the results presented here demonstrate the usefulness of N-ERC/mesothelin as a serum marker in monitoring preclinical trials for therapy in mouse and rat models of pancreas ductal adenocarcinoma: serodiagnosis in animal models can be used to enhance screening for candidate chemotherapeutic agents that could be evaluated for human use.

## Supporting Information

Figure S1 **Effect of gemcitabine in NOD-SCID mice with transplanted 634NOD cells.** The tumor weight and serum level of N-ERC/mesothelin was not decreased by treatment with 100 or 150 mg/kg gemcitabine.
(TIFF)
