# Supplementary figures and images for: Rat N-ERC/Mesothelin as a Marker for In Vivo Screening of Drugs against Pancreas Cancer
Source: PLoS One. 2014 Oct 27;9(10):e111481. doi: 10.1371/journal.pone.0111481 (PMC4210215; doi:10.1371/journal.pone.0111481)

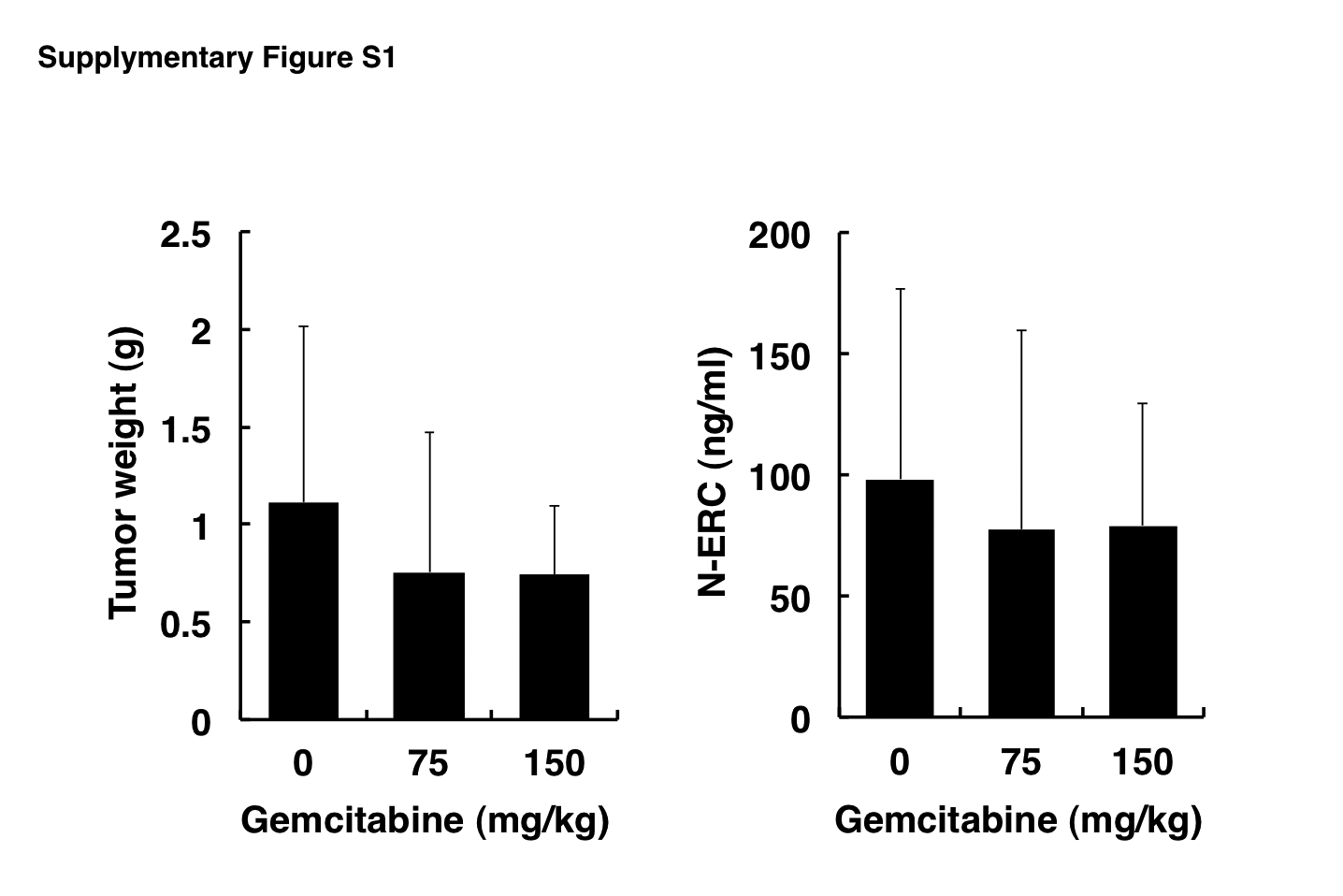

Supplement: Figure S1 — Effect of gemcitabine in NOD-SCID mice with transplanted 634NOD cells. The tumor weight and serum level of N-ERC/mesothelin was not decreased by treatment with 100 or 150 mg/kg gemcitabine. (TIFF) [file pone.0111481.s001.tiff]
